# The influence of contextual factors on the sustainability of the family talk intervention after implementation when a parent of children or youths has a life-threatening illness

**DOI:** 10.1017/S147895152510117X

**Published:** 2025-12-29

**Authors:** Ulrica Åsberg, Tina Lundberg, Malin Lövgren, Ingrid Thermaenius, Anette Alvariza, Camilla Udo

**Affiliations:** 1 School of Health and Welfare, Dalarna University, Falun, Sweden; 2 Post Graduate School for Integrated Care, Örebro University, Örebro, Sweden; 3 Department of Health Care Sciences, Marie Cederschiöld University, Stockholm, Sweden; 4 Advanced Pediatric Home Care, Astrid Lindgren Children’s Hospital, Karolinska University Hospital, Stockholm, Sweden; 5 Department of Culture and Society (IKOS), Linköping University, Norrköping, Sweden; 6 Research and Development unit/Palliative Care, Stockholms Sjukhem, Stockholm, Sweden

**Keywords:** FTI, psychosocial intervention, children or youths, contextual factors, palliative care

## Abstract

**Objectives:**

There is a lack of family-based psychosocial support interventions in palliative care when a parent of children or youths has a life-threatening illness. One intervention that has shown positive effects is the family talk intervention (FTI). This study aimed to describe the influence of contextual factors on FTI sustainability, as perceived by healthcare professionals (HCPs), after a median of 18 months of implementation in clinical practice in cancer and palliative care when a parent of children or youths has a life-threatening illness.

**Methods:**

Focus groups and individual interviews were conducted with 15 HCPs working with FTI. Data were analyzed using conventional qualitative content analysis.

**Results:**

HCPs identified contextual factors that facilitated or hindered the use of FTI. The analysis resulted in 3 categories, Trying to prioritize FTI and coordinate families in a complex context is challenging, Working alone without FTI-educated colleagues hampers sustainability, the satisfaction of seeing families become stronger contributes to a receptiveness for change.

**Significance of the results:**

This study shows that organizational support and resources, alongside the individual’s facilitating factors, such as receptiveness for change, are crucial for sustainability after the initial implementation. Witnessing a positive impact is motivational and also supports the sustainability of an intervention despite contextual constraints.

## Introduction

The family talk intervention (FTI) was implemented in clinical practice with the intention of improving psychosocial support for families when a parent of children or youths has a life-threatening illness. Implementation involves the execution of a plan, program, or intervention within a specific context. In healthcare, new evidence-based practices and guidelines are continually being integrated into daily routines to enhance patient outcomes (Hasson and von Thiele Schwarz U 2023; Public Health Agency of Sweden 2024). Sustainability is essential to ensure the lasting effectiveness of new practices and guidelines. This refers to factors that create continued use of an intervention after initial support has ended, highlighting the importance of its integration into daily routines to maintain its desired effects (Moore et al. 2017; Hasson and von Thiele Schwarz U 2023).

When implementing interventions in healthcare, it is crucial to recognize the influence of context to ensure success and long-term sustainability (Hailemariam et al. 2019; Dai et al. 2024). It is, therefore, important to study contextual factors, i.e., factors that are not part of the intervention itself, but which influence its implementation and sustainability (Pfadenhauer et al. 2015; Hailemariam et al. 2019; Damschroder et al. 2022). Several implementation frameworks highlight this importance (Cane et al. 2012; Harvey and Kitson 2016; Damschroder et al. 2022), including the Consolidated Framework for Implementation Research (CFIR). This defines contextual factors as unique circumstances in the environment in which the implementation takes place, i.e., laws, regulations, organizational structures, work environment, knowledge, behaviors, and attitudes of those using the intervention (Damschroder et al. 2009, 2022).

In palliative care, the implementation of new interventions faces challenges due to a patient’s life-threatening condition and the families’ vulnerable situation, complicating the recruitment and retention of participants in implementation studies (Chen et al. 2014; Helde Frankling et al. 2021; ES et al. 2023). At the same time, support interventions are crucial as families navigate the complexity of illness within their everyday life and parental role (Semple et al. 2021; Marshall et al. 2022; Rodriguez et al. 2023). The well-being of the patients and their families should be safeguarded by offering psychosocial support alongside care (National Board of Health and Welfare 2013; International Association for Hospice and Palliative Care 2018; World Health Organization 2023). In Sweden, palliative care is mostly provided by a multi-professional team, including physicians, physiotherapists, assistant nurses, occupational therapists, registered nurses (RNs), and healthcare social workers (HSWs) whose role also involves managing families’ psychosocial needs (Spruyt 2011; Swenurse 2019; Bitschnau et al. 2020; National Association of Social Workers 2024). If these needs are especially complex, spiritual care or mental health professionals can be consulted. However, access to evidence-based psychosocial interventions for the whole family is limited (Soikkeli-Jalonen et al. 2022; Romare et al. 2023; Yy et al. 2024).

One support intervention that includes the whole family is the FTI, first designed and evaluated in a psychiatric context (Beardslee 2002). The goals of FTI also align with the needs of families in which a parent has a life-threatening illness and requires palliative care. Although pilot studies indicate that FTI is feasible, effective, and beneficial in these settings (Alvariza et al. 2021; Eklund et al. 2021; Weber et al. 2022), it is important to ensure that its implementation in clinical practice is sustainable so that patients and their families can continue benefiting from such support (Damschroder et al. 2009; Smith et al. 2024). Since little is known about the influence of contextual factors when implementing psychosocial interventions for families in palliative care (Pakenham and Martin 2024), including FTI, this area needs more research (Dai et al. 2024). Furthermore, the continued use of an intervention, i.e., its sustainability, may be challenging (Hailemariam et al. 2019; Flynn et al. 2023). Examining contextual factors involved in implementation (Nielsen 2015) can reveal hinders or facilitators (Damschroder et al. 2022). Moreover, learning about contextual factors and their impact can facilitate the future planning of implementation strategies in similar complex intervention studies (CH et al. 2017) and an understanding of what supports sustainability. This study, therefore, aimed to describe how HCPs perceive and experience the influence of contextual factors on the sustainability of FTI after implementation in clinical practice when a parent of children or youths has a life-threatening illness.

## Methods

### Design

This study was part of a larger study evaluating FTI among families when a parent of children/youths has a life-threatening illness, using an effectiveness-implementation hybrid design (Curran et al. 2022). In the present study, focus group interviews were performed with HCPs using a qualitative inductive approach (Polit and Beck 2021).

### The family talk intervention

FTI is a manual-based family intervention. It has an eclectic theoretical framework which integrates narrative theory, dialogical theory, and psychoeducation (Beardslee 2002). In FTI, the family members exchange their stories and produce a shared family history. The goals are to facilitate family communication, increase members’ knowledge about the illness, support parenting, and make the children visible. FTI comprises 6 meetings, with extra meetings if necessary, where family members and FTI-trained HCPs meet the family members in different constellations (Table 1). The meetings focus on their reflections and thoughts about the illness and family-related issues from the parents’ and children’s perspectives.

**Table 1.** The family talk intervention: the content of the meetings and family members involved

Meeting	Family member	Content
1−2	Parents/guardians	The parents’ experiences of the situation, their view of each child’s situation, questions, and worries. The parents formulate the family’s goals of participation in FTI.
3	The child/children	Each child’s understanding of the situation, questions, and worries.
4	Parents/guardians	Summary of meeting 3 with the child/children. Planning of meeting 5 (“the family talk”).
5	The whole family	The family talk. The focus is on questions and issues raised earlier by the family members.
6	The whole family or the parents/guardians	Follow up with a focus on communication within the family to achieve the family’s goals in the future.
7−11	The whole family or parts of the family	If needed, extra meetings.

### Implementation procedure

**Figure 1.**
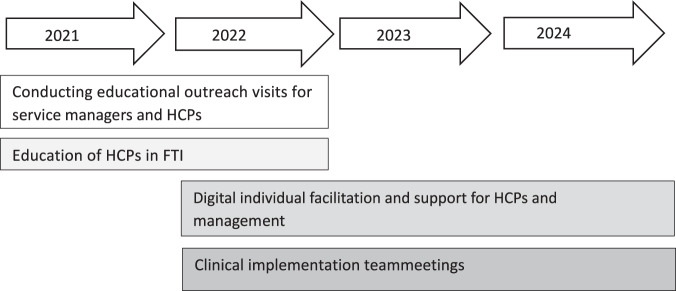
Overview of the implementation process.

The strategy for education about, and implementation of, FTI was part of a multifaceted implementation strategy (Proctor et al. 2013) including several components (Figure 1). The researchers also provided information visits to managers and HCPs at each unit, and support as external facilitators, including availability to answer questions about the study. From 2021 to 2022, HCPs participated in a 10-session FTI education program led by 2 FTI-experienced HSWs. After completing the training, the HCPs had access to weekly digital group meetings with the FTI-educators for individual supervision, if needed, focusing on FTI methodology. After the FTI education, HCPs started to offer FTI to families where a parent had a life-threatening illness and had at least 1 child aged between 0 and 24 years.

### Setting and participants

This study was conducted in an urban region in Sweden and included both specialized home care services and acute hospital wards, each with a focus on caring for adult patients with a life-threatening illness, mainly a cancer diagnosis. In Sweden, specialized home care and hospital ward services may be of different sizes depending on the healthcare organization’s area of responsibility and geographical size. Some parts of the region, therefore, have more than 1 specialized home care team. The inhabitants of the different areas vary in number and age, with some areas having an older population. Consequently, the number of families with children (age 0–24 years) varies depending on the area and affects how many families the HCPs can access in their home care teams to offer FTI.

One group of HCPs included in the present study worked in specialized home care in multi-professional teams and usually met the patient and family in the patient’s home. The other group of included HCPs worked in the hospital wards. They were also part of a multi-professional team but had a consultative role, meaning that these HCPs received referrals for patients who needed psychosocial support. The HCPs working in the hospital wards met with families in the hospital, not in the patient’s home. The specialized home care and hospital wards gave patients access to care 24 hours a day, 7 days a week.

When the managers in the eligible clinical settings were contacted and informed about the study, an agreement was also reached about the allocation of time for the HCPs to take part in the FTI-education; the researchers covered the costs of education. After the education, the HCPs were expected to use FTI as an alternative support method in their daily practice. Participants in this study were HSWs (*n*=13) and RNs (*n*=2) from 4 specialized home care services (*n* = 8) and 1 university hospital caring for patients with cancer (*n* = 7). All HCPs were women aged between 28 and 66 years (median 48 years) with work experience of between 3 and 35 years (median 8 years). Twenty-seven families started FTI during this period, of which 7 discontinued due to various factors, such as death or crisis.

### Data collection

This study included data from 3 focus group discussions (FGD) (2, 4, and 6 participants in each group). The FGD with only 2 participants was planned to include more but, for various reasons, some were unable to attend. Three individual interviews were held with 2 HSWs and 1 RN who had been unable to attend the FGDs. The FGDs were conducted by two researchers, one serving as the moderator guiding the discussions and one observing and taking notes. A discussion guide with probing questions was used to stimulate further discussion (Wong 2008), focusing on HCPs’ perceptions and experiences of working with FTI in families when a parent had a life-threatening illness. The same guide was used in the FGDs and interviews, starting with the open question “How did you feel about working with FTI in your unit.” The discussions were held digitally to facilitate participation. They were conducted between May and November 2023 when the HCPs had worked with FTI for up to 20 months (median 18 months, minimum 5 months). The FGDs lasted between 38 and 68 minutes (median 67 minutes) and the individual interviews between 26 and 42 minutes (median 27 minutes). Participants seemed to find the subject of providing support to families important, so they engaged easily in the discussions, making the data rich. All FGDs and interviews were recorded and transcribed verbatim.

### Data analysis

The data analysis was guided by Hsieh and Shannon’s (2005) conventional content analysis approach, since this method is suggested to be well-suited to describing healthcare contexts and subjective perspectives. The analysis process was not linear, but instead moved between the whole and the parts, keeping close to the original text. Initially, the first and last authors carried out a naïve reading of the transcripts to gain a sense of the whole before discussing their first impressions. The first author then continued the analysis by identifying and condensing meaning units, which were coded with descriptive codes, i.e., short descriptive labels of the content of each meaning unit. All codes were then thoroughly discussed by the first, second, and last authors, after which they were somewhat revised before being grouped according to similarities. The codes were then abstracted further into preliminary subcategories and categories by the first author before being discussed with the second and last authors. The categories were then revised before being checked thoroughly against the original statements in the interviews. A joint discussion between all authors was held and, when consensus was reached, the analysis was considered complete.

### Ethical consideration

The Swedish Ethical Review Authority ethically approved the study (Dnr. 2020-06340, Dnr. 2021-04686 and 2022-01949-02, 2023-02389-02). The HCPs received written and oral information about the study and were invited to participate in accordance with the principles of the Declaration of Helsinki (World Medical Association 2013).

## Results

The analysis resulted in 3 categories of perceptions and experiences: trying to prioritize FTI and coordinate families in a complex context is challenging; working alone without FTI-educated colleagues hampers sustainability; and the satisfaction of seeing families become stronger contributes to a receptiveness for change. The results for each category are further described below and exemplified with interview quotations.

### Trying to prioritize FTI and coordinate families in a complex context is challenging

This category illustrates HCPs’ perceptions of contextual factors in their work environment, when working with FTI.

HCPs highlighted challenges in prioritizing their work with FTI due to limited resources and complex family coordination. Time constraints and lack of FTI-educated colleagues hindered planning, while the need for family-friendly environments complicated coordination and scheduling flexibility. HCPs in the acute hospital ward expressed challenges in getting the children to the hospital, since the environment and office-hours meetings were not adapted the children’s needs and daily routines. To ensure access to FTI, HCPs conducted sessions in various environments, including hospitals, in patients’ homes, and digitally. While digital meetings worked well for adults, younger children often struggled to maintain attention. The HCPs, therefore, considered home-based FTI to be more beneficial in offering a comfortable environment for children.

*It makes a big difference when you go home to the family and talk to the children in their own environment and on their own terms, you can talk for a while, you can go out, you can look at their room, you can do things like that … so … I’d rather work with this … at home with families, I can say that.*

HCP, hospital

HCPs recognized the benefits of FTI for families where a parent faces a life-threatening illness, but worried that FTI’s time-intensive nature limited their ability to assist other patients. They noted that preparation and follow-up was particularly demanding for larger families, causing hesitation in offering FTI due to resource constraints, such as tight schedules. HCPs felt pressured into justifying prioritizing FTI over taking on new families, fearing that manager’s cost concerns might affect the continued use of FTI.

*But on the other hand, I think managers tend to be more worried about it because it takes too long, … that it needs more of us … that’s their focus and if it turns out that way, they [the managers] will maybe …, try to find ways to avoid us needing to work with the families this way.*

HCP, specialized home care

Determining the right time to offer FTI was considered challenging. While some families were receptive early in the illness, others became available later, leading to delays and increased waiting lists, further complicating prioritization. Early patient contact was suggested to better assess families’ unique needs and plan for FTI. However, competing priorities, such as medical appointments, often took precedence, making planning complicated. Balancing the complexity of patients’ illnesses, and taking all medical appointments into account alongside the family’s needs, required, therefore, constant adjustments.

*I think that the medical [perspective] has often taken priority, so it has been a bit difficult. There have been several [families] that I have offered [FTI] to who have declined due to ongoing treatment. They’ve said that they’ve already been at the hospital so much…*

*HCP, hospital*

### Working alone without FTI-educated colleagues hampers sustainability

This category highlights the HCPs’ feelings of managing FTI alone and their experience of receiving support while working with FTI.

HCPs explained that while they were accustomed to working independently in clinical care, providing FTI often left them feeling isolated and solely responsible for the task. The sense of being alone was particularly challenging and described as a hindrance, especially when they lacked FTI-trained colleagues with whom they could discuss and reflect.

*But as I said, when there’s no one else working with it [FTI], you’re a bit alone in the group and … supervising this in the group is not much fun. Basically, there are not that many people who see families or who work with it*.

HCP, hospital

The support and commitment from FTI-trained colleagues and the multi-professional team were seen as crucial in enabling the HCP’s work with FTI. Regular meetings with FTI-trained colleagues were valuable for sharing ideas and receiving encouragement. When the multi-professional team understood the HCPs’ needs for time with patients, it alleviated the feeling of being alone and strengthened their motivation and willingness to continue using FTI.

*They [the team] have been very understanding – if I spend more time with a patient and document that it’s for FTI, no one questions that … that is … that way it works well*.

HCP, specialized home care

Managerial support also played a crucial role. The active engagement of managers fostered trust and allowed the HCPs the freedom to plan their work, making them feel supported and less alone. Conversely, a lack of interest increased their sense of isolation.

### The satisfaction of seeing families become stronger contributes to a receptiveness for change

This category highlights how the HCPs’ work with FTI broadened their understanding of the children’s and family’s perspectives, increased the HCPs receptivity to the children’s positions, and contributed to motivation. Seeing families empowered through FTI motivated the HCPs to adapt their approach and continue using FTI. Witnessing the positive impact on families fostered gratitude and well-being among the HCPs, reinforcing the meaningfulness of their work.

*It feels great to have it [FTI]. To have it within your [competence]… yes to know that you can have such conversations, it feels good … It has strengthened me.*

HCP, specialized home care

Seeing the transformation in families increased the HCPs’ self-confidence and sense of competence, making them feel more comfortable when engaging with children in conversations, which in turn created a positive feedback loop that encouraged them to embrace FTI. The HCPs noted that their work with FTI also contributed to openness and commitment within the multi-professional team, since they recognized the benefits for the families, leading to a stronger focus on addressing the needs of both families and children.

This was explained as crucial in expanding the use of FTI and strengthened the HCPs’ motivation to continue working with it, despite difficulties, believing it would benefit more families in the future.

*… that … like the team becomes more aware of … informing me that there’s a family with children on the way in. Nowadays, I often find out … even before they’re registered … that now we’re enrolling a family with children… so that I can allocate time and get involved*

HCP, specialized home care

HCPs emphasized that using FTI allowed the inclusion of the children’s perspectives, something that was previously challenged by the healthcare services’ adult-centric focus. Shifting from a narrow adult-focus made them more satisfied with their work and more dedicated to advocating for children’s rights.

*… that children are even welcome to see us … that you don’t just say…. think this way and like that, to the parents … and then you maybe … contact the school counselor… so that they are actually being offered something here.*

HCP, hospital

## Discussion

This study describes how HCPs perceived and experienced the influence of contextual factors on the sustainability of FTI after a median of 18 months of implementation in clinical practice among families where a parent of children or youths was facing life-threatening illness. The results show that contextual factors play a dual role, serving both to hinder and facilitate. Contextual factors that hindered included FTI staff shortages, lack of facilities, and competing patient and family needs while managing FTI. Working without FTI-educated colleagues meant that the HCPs had no one to discuss FTI with, leaving them with a sense of being alone. Although working with FTI was considered positive, concerns were raised about whether the organization would support the continued use of FTI. Simultaneously, support from the manager and witnessing the positive impact of FTI on families could facilitate sustainability. Interestingly, results in this study show that conducting FTI in the family’s home instead of in the hospital environment was perceived by HCPs as a facilitating contextual factor since it was possible to, for example, spontaneously take a break if needed and move around in the home. The satisfaction derived from seeing families become stronger increased the HCPs’ motivation to continue using FTI despite obstacles and provided a deeper insight into children’s needs among the HCPs and their colleagues in the multi-professional team, which in turn allowed more families to receive FTI.

The contextual factors described by HCPs align with the CFIR framework, highlighting the importance of resource availability and organizational support (Damschroder et al. 2009, 2022). This study indicates that insufficient resources may hinder the sustainability of new interventions, which is consistent with prior research (Adams et al. 2021). Addressing resource shortages is important since it can negatively impact the quality of care and support. Furthermore, it risks creating ethical dilemmas for professionals who face tough decisions on support options amid constraints, including lack of proper interventions to offer families. Such challenges underscore the urgent need to address the resource shortage to ensure that HCPs can stay equitable and ethical (Adams et al. 2021). This extra stress was visible in the study’s results when HCPs explained that they must make difficult choices when providing optimal support in a context with resource constraints. Furthermore, the HCPs in this study expressed concerns that managers might see FTI as too resource-intensive, leading to worries that FTI could be rejected as a form of support offered to families. According to Gustavsson and Tinghög (2020), cost-effectiveness is an ongoing process in healthcare organizations that affects the resources available, which can create stress for healthcare professionals (HCPs). Moral stress can arise when experiencing that the best care and support for patients conflicts with what is best for the organization (Hossain and Clatty 2021). This can lead to professional dissatisfaction and people leaving their professions due to the inability to deliver care and support in line with their values (Tamata and Mohammadnezhad 2023). Although it is also the HCPs responsibility to make sure that allocated resources are used in a way that is most beneficial for the patient and family, management is identified by CFIR as a key contextual factor (Damschroder et al. 2009, 2022) for creating proper working conditions (Barasa et al. 2015) where evidence-based support interventions can be sustainable.

The HCPs in this study highlighted how medical treatments often take priority over other interventions, such as FTI, hindering planning and execution. If psychosocial support is frequently considered secondary to physical treatments, then this reflects broader systematic issues (Rodin 2018; Taels et al. 2021; Liang et al. 2024), even though modern palliative care has a multidisciplinary approach (Connor 2020). Finding strategies to increase the integration of psychosocial support and medical care becomes, therefore, important in supporting the patient and the family, since studies show that psychosocial interventions for families with life-threatening illnesses have a significant effect (Weber et al. 2022; Liang et al. 2024).

This study showed that HCPs’ often felt alone when supporting families with FTI, which aligns with Yan et al. (2024) who found that staff with unique tasks can easily feel alone in their work situation. They define workplace loneliness as a negative emotion experienced by employees when lacking social interaction and support within the organization, including from the manager. CFIR highlights leadership as an important contextual factor since managers can empower individuals and teams within the organization during the implementation processes. However, the behavior, experience, and abilities of the staff are also key contextual factors when implementing a new method (Damschroder et al. 2009, 2022). This study shows that it is not only the manager’s support that is a vital contextual factor for successful implementation, but also seeing positive effects, such as strengthening families and making the children’s perspectives visible. This is in line with Weick et al. (2005) who believe that beneficial changes can take place in healthcare when HCPs witness patients’ positive changes.

## Conclusion

This study highlights the dual nature of contextual factors, which can both hinder and facilitate the sustainability of a psychosocial support intervention, such as FTI, after implementation. A key insight is the sense of inner satisfaction that HCPs may experience when witnessing positive family development from participating in FTI, serving as a strong counterbalance to contextual barriers. Additionally, organizational support and resources, alongside the individual’s facilitating factors, such as receptiveness for change, are crucial for sustainability after the initial implementation has ended. However, attention must be given to resource availability and funding to ensure new interventions do not compromise but instead refine and improve the standard of care and support. FTI could ideally be included in standard care as one of several evidence-based psychosocial support interventions to meet the needs of families. FTI may be a means of refining and structuring, rather than replacing, existing support.
